# Nosocomial transmission of *Clostridium difficile* Genotype ST81 in a General Teaching Hospital in China traced by whole genome sequencing

**DOI:** 10.1038/s41598-017-09878-8

**Published:** 2017-08-29

**Authors:** Juanxiu Qin, Yingxin Dai, Xiaowei Ma, Yanan Wang, Qianqian Gao, Huiying Lu, Tianming Li, Hongwei Meng, Qian Liu, Min Li

**Affiliations:** Department of Laboratory Medicine, Renji Hospital, School of Medicine, Shanghai Jiaotong University, Shanghai, China

## Abstract

*Clostridium difficile* infection (CDI) is increasingly recognized globally as a cause of significant morbidity and mortality. This study aimed to provide insight into the various dynamics of *C. difficile* transmission and infection in the hospital. We monitored the toxin and resistance profiles as well as evolutionary relationships of *C. difficile* strains to determine the epidemiology over time in a teaching hospital in Shanghai, China between May 2014 and August 2015. The CDI incidence of inpatients and outpatients were 67.7 cases and 0.3 cases per 100,000 patient-days, with a nosocomial patient-environment-patient transmission in May and June 2015. *C. difficile* genotype ST81, a clone with *tcdA*-negative and *tcdB*-positive, was not only the most common strain (30.8%, 28/91) but also had much higher resistance rates to clindamycin and moxifloxacin compared with non-ST81 genotypes. Hospitalized patients infected with ST81 genotypes were over 65 years of age and had more comorbidities, however patients infected with ST81 presented with less clinical symptoms than non-ST81 infected patients. This study provides initial epidemiological evidence that *C. difficile* ST81 is a successful epidemic genotype that deserves continuous surveillance in China.

## Introduction

*Clostridium difficile* is an anaerobic, gram-positive, spore-forming bacterium, found as a commensal or pathogen in the intestinal tracts of many species of animals and in the environment^1^. *C. difficile*-associated infection (CDI) is not only a leading cause of antibiotic-associated diarrhea but also an important nosocomial infection that causes a spectrum of diseases ranging from asymptomatic carriage or mild diarrhea to severe life-threatening pseudomembranous colitis or even death^2, 3^. Morbidity and mortality from CDI have increased significantly over the past 10 years, making *C. difficile* as one of the most problematic emerging pathogens worldwide^4–7^.

Unlike other common health-associated pathogens, *C. difficile* produces highly resistant and transmissible spores that resist infection control measures. CDI generally requires a combination of pathogen acquisition, risk factors, and host susceptibility^8^. Systematic surveillance of a combination of *C. difficile* epidemiology, transmission, and host susceptibility is not routinely performed in China.

We conducted a study of CDI in a general teaching hospital in China over a 16-month period between May 2014 and August 2015 for insight into the various dynamics of *C. difficile* transmission and infection in the hospital environment. In May and June 2015 a major outbreak of *C. difficile* was detected, and investigations by whole genome sequencing were undertaken. Here we present the results and lessons learned from the outbreak investigation.

## Results

### *C. difficile* genotype ST81 was the predominant clone causing CDI

To obtain a complete overview of the epidemiology of *C. difficile* infection, 91 isolates from 798 unformed stool samples collected between May 2014 and August 2015 were analyzed by MLST. There were a total of 80 nonduplicate toxigenic *C. difficile* isolates, excluding 11 non-toxigenic isolates, making The CDI incidence of inpatients 67.7 cases per 100,000 patient-days and 0.3 cases per 100,000 patient-days in outpatients. 80 toxigenic isolates and 11 nontoxigenic isolates were classified into 19 and 6 STs, respectively (Table 1). *C. difficile* genotype ST81 was identified corresponding to 30.8% of all strains (28/91) and was the predominant clone causing CDI (28/80; 35.0%), followed by ST2 (9.9%; 9/91), ST54 (8.8%; 8/91), and ST129 (8.8%; 8/91). ST3 strains included both toxigenic and non-toxigenic types. None of the isolates contained *ctdA* and *ctdB*. Of the 80 cases, 44 cases (55.0%) occurred in female patients and 17 cases (21.3%) occurred in outpatients. Specimens were also tested for *S. aureus, Salmonella* and *Shigella*, but these bacteria were not found. Detail information of 91clinical isolates are shown in Supplementary Table S1.

**Table 1:** Genotypes of *C. difficile* isolated from clinical patients between May 2014 and August 2015.

	Genotypes	Toxin genes	Number	%
CDI(n = 80)	ST81	A−B +	28	30.8
	ST2	A + B +	9	9.9
	ST54	A + B +	8	8.8
	ST129	A + B+	8	8.8
	ST3	A+B+	6	6.6
	ST37	A−B+	2	2.2
	ST319^a^	A+B+	1	1.1
	Others^b^	A+B+	18	19.7
No-CDI(n = 11)	ST39	A−B−	5	5.5
	ST26	A−B−	2	2.2
	ST320^a^	A−B−	1	1.1
	ST259	A−B−	1	1.1
	ST3	A−B−	1	1.1
	ST48	A−B−	1	1.1
	Total	−	91	100

A + B + : toxin A-positive, toxin B-positive strain; A−B + : toxin A-negative, toxin B-positive strain; A−B−: toxin A-negative, toxin B-negative strain.

^a^Newly identified STs in this research.

^b^ST35, ST98, ST8, ST14, ST256, ST102, ST139, ST28, ST42, ST51, ST55 and ST17.

**Figure 1:**
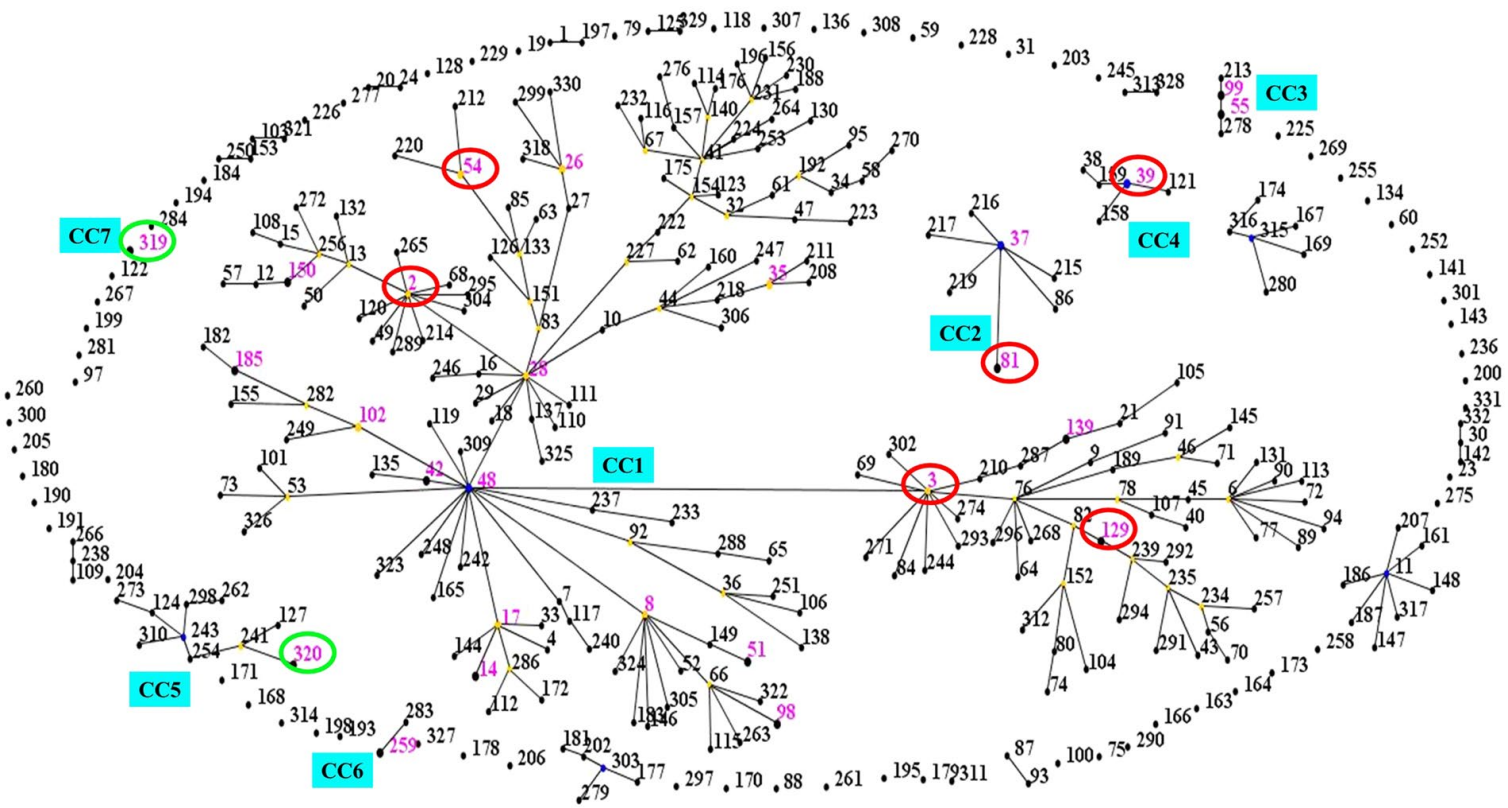
eBURST analysis of *C. difficile* using all STs available in the MLST database as of January 2016. ST nodes: blue: primary founder; yellow: sub-group founder; purple: STs identified in this research; green: newly identified STs in this research; red: >5 STs in this research; Cyan: clonal complex groups (CC).

The eBURST analysis of *C. difficile* using all STs available in the MLST database is shown in Fig. 1 to analyze the evolutionary and genetic diversity. The eBURST algorithm clustered all 26 STs isolated from clinical patients into CC1 (51/91; 56.0%), five minor CCs (CC2, CC3, CC4, CC5, and CC6: 39/91; 42.9%), and one single CC: CC7 (1/91; 1.1%). Two strains with new combinations of allelic profiles were assigned novel STs, ST319 and ST320. They respectively belonged to CC7 and CC5. The large cluster of isolates, ST81 with *tcdA*-negative and *tcdB*-positive, belonging to CC2, was the predominant clone causing CDI.

### *C. difficile* ST81 isolates were detected in the majority of departments and had a suspected outbreak in May and June 2015

**Figure 2:**
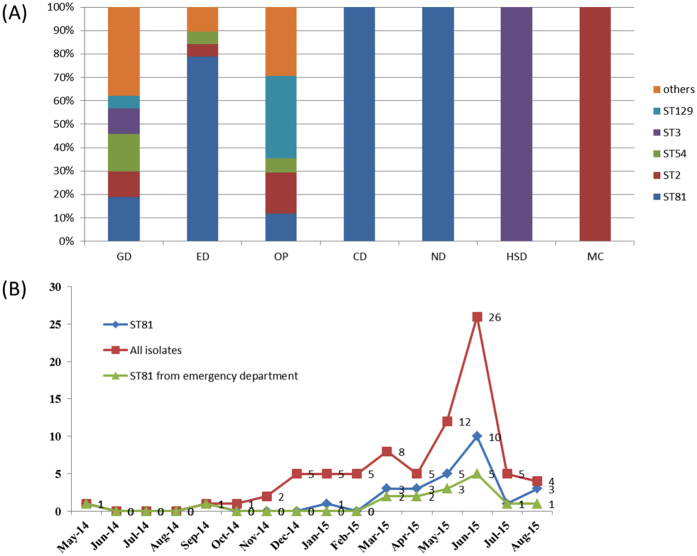
Distribution of genotypes over time by hospital departments. (**A**) Distribution of genotype according to admission departments. GD: Gastroenterology department; ED: Emergency department; MO: Medical oncology; ND: Nephrology department; OP: Outpatient; HSD: Hepatobiliary surgery department CD: Cardiology department. (**B**) Monthly distribution of *C. difficile* isolates.

A histogram of genotype distribution by hospital department is shown in Fig. 2A. Different departments exhibited different composition of *C. difficile* genotypes. The highest number of *C. difficile* (genotype and amount), was isolated from the gastroenterology department, followed by the emergency and outpatient departments. ST81, ST54, ST2, and ST3 were detected from at least three departments, suggesting that certain departments harbored endemic strains. A total of 78.9% and 18.9% of isolates from the emergency and gastroenterology departments, respectively, were ST81. One isolate from the cardiology department and two isolates from the nephrology department were ST81, which was not only the most common (28 isolates) but also detected from majority of the departments in inpatients.

The monthly distribution of all isolates, ST81 isolates, and ST81 isolates from the emergency department is shown in Fig. 2B. CDI case numbers showed an increasing trend, peaking in June 2015 due to a special genotype epidemic event, and falling to a pre-epidemic level following the outbreak. ST81 strains were isolated at rate of 1.8 per month, and an outbreak was suspected in May and June 2015 (five isolates in May and 10 isolates in June, two-fold higher than 1.8 per month). ST81 isolated from the emergency department might contribute greatly to the epidemic event in May and June 2015.

### *C. difficile* ST81 had a nosocomial patient-environment-patient transmission in May and June 2015

**Figure 3:**
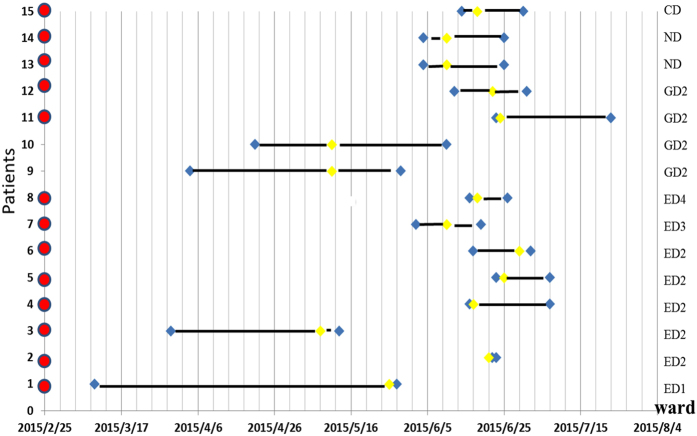
Temporal graph of 15 isolates of ST81 isolated from 15 patients (1–15). Red: The patient had undergone emergency medical treatment in the department observation room (ED0); Yellow: detection time; Blue: admission time and discharge time; Bold black line: period of hospitalization; ED1,ED2,ED3, and ED4: Emergency department wards; GD2: Gastroenterology department ward; ND: Nephrology department.

Figure 2B highlighted the potential contribution of the outbreak of ST81 to the epidemic event in May and June 2015; we next investigated the spread of 15 *C. difficile* ST81 isolates during this period, particularly in the emergency department. Of 15 infected patients, eight were from the emergency department (patients: 1,2,3, 4, 5,6,7,8), four from the gastroenterology department (patient: 9,10,11,12), two from the nephrology department (patient: 13,14) and one from the cardiology department (patient: 15). The emergency department includes a special emergency department observation room 0 (ED0) and the following four wards: ED1, ED2, ED3, and ED4. Five of eight patients in the emergency department were from ED2. The other three were in ED3 (one isolate) and ED4 (two isolates). They were all first admitted to ED0 and then to their ward. Five of the remaining seven patients were admitted to ED0 before being moved to other wards. Two gastroenterology department patients (9 and 10) did not pass through ED0. Temporal graph of 15 isolates of ST81 isolated from 15 patients is shown in Fig. 3. Ward-based network analysis showed a high level of transmission within ED0, so we examined the ED0 ward environment in the middle of June 2015, from which two ST81 isolates were isolated from toilets (named 73 and 74).

**Figure 4:**
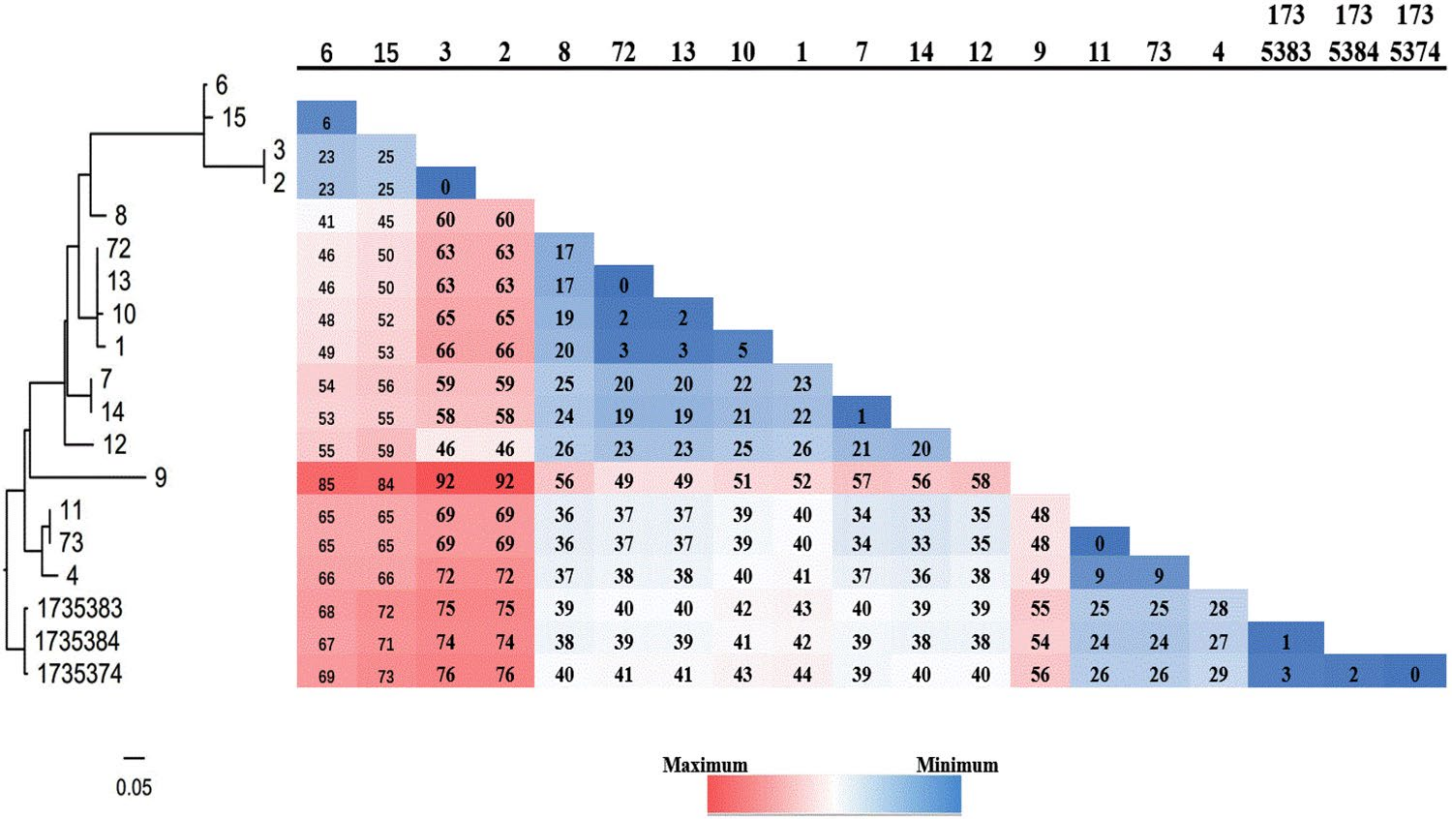
Analysis of phylogenetic tree and SNPs within *C. difficile* genotype ST81 in May and June 2015. Isolates 73 and 74 were separated from the ED0 environment in early June**;** SRR 1735383, SRR 1735384 and SRR1735374 were three annotated ST81.

Whole-genome sequencing has a higher discriminatory power for investigations of nosocomial transmission. 16 isolates of *C. difficile* genotype ST81((representing 14 isolates from the patients and two from environment) were sequenced and complete chromosome sequences. The 16 isolates and three annotated ST81 genomes (SRR 1735383, SRR 1735384 and SRR1735374) were subjected to the CSI Phylogeny 1.4 server in order to investigate their single-nucleotide polymorphisms (SNPs)-based phylogeny. The sequences were aligned to the reference chromosome of isolate 73 and then SNPs were called. Results of WGS analysis of *C. difficile* isolates from patients and environment are shown in Fig. 4. According to a former study in which 0–2 SNVs were identified between transmitted isolates obtained less than 124 days apart, the same ST came from the same origin^9^. Isolates 72 and 73 were all separated from the ED0 environment in early June. We discovered that there was only 0 SNP difference between isolates 72 and 13, 73 and 11, patients 13 and 11 were all first admitted to ED0 during this period and then to their wards, illustrating their close relationship were from environment to patient transmissions. Three isolates of ST81 (11,13 and 10) presented two or five SNPs, however, they did not exist in the same ward and hence couldn’t spread via patient to patient transmissions. Interestingly, isolates 72 and 1 presented a genetic distance of 3 SNPs. Patient 1 had been to the ED0 before the environmental epidemiologic investigation and was a potential sources of *C. difficile* transmissions since the patient may have excreted ST81 *C. difficile* into the EDO environment and contaminated other patients (11,13 and 10). In addition, we found that there were only two SNP differences between isolates 72 and 10, but this patient had never been to ED0. There may be a potential route of transmission through hospital workers or other patients. Three annotated ST81 (SRR 1735383, SRR 1735384 and SRR1735374) presented zero or three SNPs, illustrating their close relationship which is same as a former study^10^. The remaining cases had sufficient genetic diversity (>10 SNVs from any previous cases) to represent transmissions originating from sources other than cases that were included in the study.

Unit-based nurse leadership and physicians were notified of patients identified with CDI and needed to understand the hazards and precautions of *Clostridium difficile* infection. These departments were cleaned daily with 1,000 ppm available chlorine. In emergency department, patients with diarrhea needed extra attention. Those who visited and cared for these patients were encouraged washing hands with soap and water. The toilets which were potential environmental reservoirs should be cleaned with at least 1,000 ppm available chlorine so as to prevent cross infection. The number of CDI cases then showed a decreasing trend in July and August 2015.

### Compared with non-ST81 genotypes, ST81 genotype has significantly less toxin production but similar spore quantities

**Figure 5:**
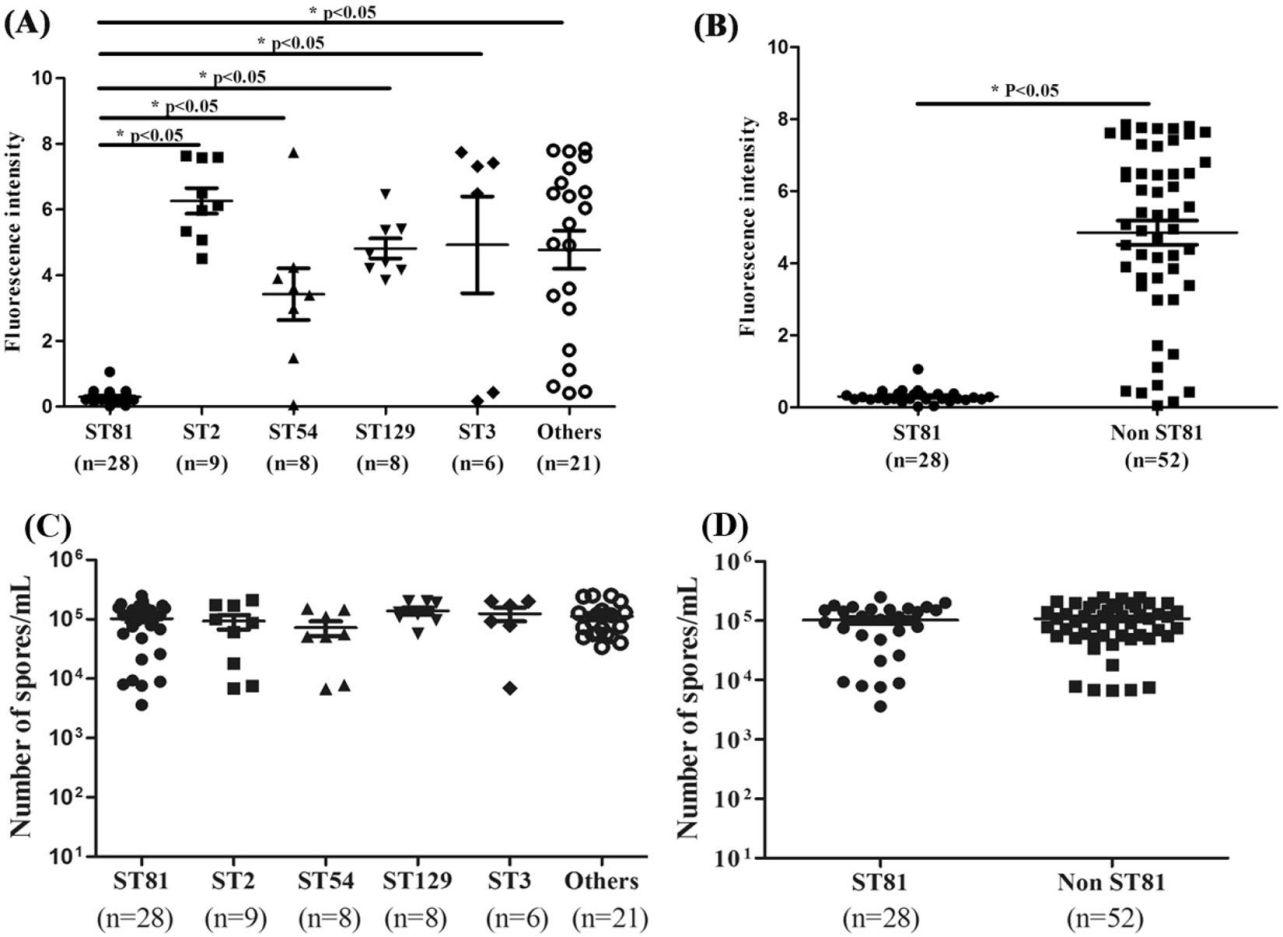
Comparison of toxin production and Sporulation determination by clinical toxigenic *C. difficile* genotypes. The toxin production and sporulation determination data are shown as the means ± SEM. The genotypes with significant differences were marked with *P < 0.05. (**A**) A comparison of toxin production among isolates with different genotypes: ST81, ST2, ST54, ST129, ST3, and others. (**B**) A comparison of the toxin production of the isolates with ST81 genotype and non ST81 genotypes. (**C**) Comparison of the sporulation determination of the isolates with different genotypes: ST81, ST2, ST54, ST129, ST3, and others. (**D**) Comparison of Sporulation determination of the isolates with ST81 genotype and non ST81 genotypes.

As shown in Fig. 5A,B, clinical toxigenic *C. difficile* isolates with different genotypes may have different abilities to product toxins. The toxin production of epidemic ST81 isolates (0.3 ± 0.2) was less than that in other groups of genotypes 6.3 ± 1.2 of ST2, 3.4 ± 2.2 of ST54, 4.8 ± 0.9 of ST129, 4.9 ± 3.6 of ST3, 4.8 ± 2.7 of others. Compared with non-ST81 genotypes, ST81 genotype had significantly less toxin production (0.3 ± 0.2, p < 0.05). The significantly reduced toxin production of ST81 genotype could be explained by its toxin type (*tcdA*-negative and *tcdB*-positive). As shown in Fig. 5C,D, no significant difference was detected between the sporulation determination of the five groups. Compared with ST81 genotype and non ST81 genotypes, similar spore quantities were obtained (1.0 × 10^5^) ± (0.9 × 10^5^) vs. (1.1 × 10^5^) ± (0.9 × 10^5^), respectively.

### Compared with non-ST81 genotypes, ST81 genotype had a much higher resistance rate to clindamycin and moxifloxacin

We categorized the isolates as ST81 isolates and non-ST81 isolates and compared the antibiotic resistance in two groups. Clindamycin resistance of ST81 isolates reached 71.4% compared with 48.1% of non-ST81 isolates (P = 0.03), and the frequency of resistance to moxifloxacin was five-fold higher in the large-cluster isolates than in the other isolates (67.9% and 13.5%, respectively, P < 0.01). Five ST81 isolates were resistant to tetracycline; however, non-ST81 isolates were all susceptible to tetracycline (Table 2). Compared with non-ST81 genotypes, ST81 was not only the most common isolate but also had a much higher resistance rate to clindamycin and moxifloxacin. Antimicrobial susceptibility testing of all toxigenic *C. difficile* strains are in Supplementary Table S2.

**Table 2:** Antibiotic Resistance among ST81 and non-ST81 isolates.

**Drug Agent**	**No. (%) Resistant Isolates**		**P**
	**ST81(n = 28)**	**Non ST81(n = 52)**	
CLI	20(71.4)	25(48.1)	P = 0.03
MOX	19(67.9)	7(13.5)	P < 0.01
TET	5(17.9)	0	NA
MET	0	1	NA

CLI: clindamycin; MOX: moxifloxacin; TET: tetracycline; MET: metronidazole

NA: not applicable.

### Compared with ST81 genotypes, hospitalized patients infected with ST81 genotypes were over 65 years of age and had more comorbidities

17 isolates from outpatients and four isolates from emergency observation room patients without detailed medical records were excluded from study, leaving 25 ST81 isolates and 34 non-ST81 isolates for comparison of clinical characteristics. Age and gender was not different between excluded and included patients (Supplementary Table S3) and clinical information of hospital patients infected with ST81 and non-ST81 C. difficile are shown in Supplementary Table S4. As shown in Table 3, patients infected with ST81 isolates were significantly older than those infected with non-ST81 isolates. Mean age of patients with ST81 isolates and other genotypes was 68.4 and 47.0 years respectively (P < 0.01). Of patients infected with *C. difficile* ST81, 60.0% were older than 65 years compared with 20.6% of non-ST81 infected patients. Charlson Comorbidity Index was higher in patients with *C. difficile* ST81 infection than in those infected with non-ST81 isolates (2.8 ± 2.1 vs. 0.7 ± 1.0). Of patients with *C. difficile* ST81 infection, 48.0% had more than three comorbidities compared with 2.9% of patients with non-ST81 infection. There were no significant differences in gender, period of hospitalization, PPI use, numbers and categories of antibiotics used, and several laboratory indexes such as serum albumin, serum creatinine and total bile acid.

**Table 3:** Demographic and clinical characteristics of hospital patients infected with ST81 and non-ST81 *C. difficile*.

Variable	ST81 Strains (n = 25)	Non-ST81 Strains (n = 34)	p value
Age(years;mean ± standard deviation[SD])	68.4 ± 15.7	47.0 ± 21.2	<0.01^a^
No. of cases > 65 years-old	15(60.0)	7(20.6)	0.03^b^
Gender			
Male	10(40.0)	20(58.8)	0.19^b^
Females	15(60.0)	14(41.5)	
period of hospitalization (days, mean ± SD)	31.5 ± 21.4	26.2 ± 24.3	0.39^a^
Serum albumin(g/L, mean + SD)	31.8 ± 5.8	35.1 ± 9.8	0.14^a^
Serum creatinine(μmol/L, mean + SD)	109.7 ± 114.4	68.2 ± 36.7	0.06^a^
Serum total bile acid(μmol/L, mean + SD)	19.3 ± 40.4	13.4 ± 34.6	0.55^a^
Charlsoncomorbidity index(mean + SD)	2.8 ± 2.1	0.7 ± 1.0	<0.01^a^
No. of cases. Scoring 0–2 (%)	13(52.0)	33(97.1)	<0.01^c^
No. of cases scoring≥3 (%)	12(48.0)	1(2.9)	
No. of antibiotics used (mean + SD)	1.8 ± 0.9	1.1 ± 1.0	0.07^a^
cephalosporins	20(80.0)	17(50.0)	0.29^b^
Fluoroquinolones	17(68.0)	16(47.1)	0.12^b^
Penicillin derivatives	10(40.0)	4(11.8)	0.16^c^
Macrolides	0	1(2.9)	NA
Glucocorticoid drugs	7 (28.0)	8(23.5)	1^b^
Fever	7(28.0)	3(8.8)	0.07^c^
Serum albumin(<20 g/L)	2(8.0)	2(5.9)	1^c^
WBC count(>12 × 10^9^ cells/L)	3(12.0)	5(14.7)	1^c^
Presence of fecal leukocytes	1(4.0)	9(26.5)	0.03^c^
Presence of fecal erythrocytes	1(4.0)	7(20.6)	0.12^c^
Presence of fecal occult blood	2(8.0)	16(47.1)	<0.01^c^

Data are no. (%) of patients, unless otherwise indicated.

^a^: The two-tailed unpaired Student’s t-test; ^b^: Chi-square; ^c^: Fisher exact tests.

NA: not applicable.

For cases diagnosed with severe diarrhea, we compared fever, serum albumin (<20 g/L), WBC count (>12 × 10^9^ cells/L), fecal occult blood, presence of fecal erythrocytes, and leukocytes for the ST81 and non-ST81 groups. Fever, serum albumin (<20 g/L), WBC count (>12 × 10^9^ cells/L) were not different between the two groups. However, the non-ST81 group had a higher rate of fecal occult blood and fecal leukocytes than the ST81 group (24.7%vs. 4%, p = 0.03 and 47.1% vs. 8%, p < 0.01, respectively). These findings suggest that clinical symptoms were less serious in patients infected with ST81 isolates than those with non-ST81 infection.

## Discussion

Currently, longitudinal and systematic data on the status of CDI in China are largely missing or uncertain. We investigated all CDI cases that occurred in a general teaching hospital in China over a period of sixteen months between May 2014 and August 2015, to supervise the epidemic trend of this special pathogen. In our study, The CDI incidence of inpatients and outpatients were 67.7 cases and 0.3 cases per 100,000 patient-days. A special hospital-endemic *C. difficile* genotype ST81 was defined, isolated from a large proportion of all toxigenic isolates (35%, 28/80), and caused an outbreak in a specific period.

It is reported that the major clone of *C. difficile* isolates in China and many other Asian countries is ST37^11–16^. Both *tcdA*-negative and *tcdB*-positive genotypes show higher drug resistance than other genotype isolates. The evolutionary analysis by MLST showed that ST81 is a one allelic *atpA* variant of ST37. It is the most common isolate among antibiotic-associated diarrhea in the hospital in shanghai, China between August 2012 and July 2013^17^. One study from Japan also found seven ST81 isolates that showed high-level resistance to clindamycin and ciprofloxacin. They reported that a genetic shift from ST37 to ST81 occurred in the hospital under study^14^. In our research, the epidemic *C. difficile* clone ST81 had a much higher resistance rate to clindamycin and moxifloxacin than non-ST81 isolates, a similar result to the Japanese research. It is reported the most common *C. difficile* genotype merging in the past decade in North America and some areas of Europe was ST1/ ribotype 027(RT027)^18^, but in many other Asian countries such as Japan, South Korea, Singapore, and China, RT027 was reported only sporadically^19–22^. ST1/RT027 was not found in our study, demonstrating that epidemiological changes of CDI varied depending on geography.

ST81 was not only the predominant genotype isolated from the CDI patients, but also detected from the majority of the hospital departments. In our study, the number of CDI cases presented an increasing trend, peaking in June 2015 with the occurrence of a special genotype epidemic event. We identified, via MLST and whole genome sequencing, that ST81 clone outbreak contributed considerably to the epidemic event in May and June 2015. According to criteria standards by MLST: most of the putative transmissions identified occurred shortly (<1 wk) after the onset of symptoms, with few >8 wk. Most incubation periods were <4 wk, with few >12 wk^23^. We couldn’t distinguish the spread of *C. difficile* ST81 genotype between patients and the environment in such short time. Interestingly, whole-genome sequencing of *C. difficile* is exquisitely sensitive. There was a nosocomial patient-environment-patient transmission in May and June 2015 in term of a former study in which 0–2 SNVs were identified between transmitted isolates obtained less than 124 days apart^9^.

Why was there nosocomial transmission of *C. difficile* genotype ST81? As we know the success of an epidemic clone relies on its transmission capacity. However, no significant difference was detected between ST81 genotype and non-ST81 genotypes in their sporulation determination. ST81 genotype, a combination of low virulence and high antibiotic resistance resisting the antibiotic pressure, was likely to favor host survival and, consequently, transmission to new susceptible hosts. As we known, overuse of antibiotics actually increased the antibiotic pressure and risk for CDI. Compared with non-ST81 genotypes, ST81 genotype had a much higher resistance rate to clindamycin and moxifloxacin. We investigated the use of clindamycin and quinolones in the emergency department. Figure S1 showed that monthly distribution of Antibiotics Use Density (AUD) for quinolones in the emergency department, there was no apparent rise in May and June 2015 and clindamycin was infrequently used in the ward. Several systematic review had evaluated the role of different antibiotics but much of the literature was different^24^. There would be other drug selective pressures in our hospital. Moreover, patients infected with ST81 isolates were significantly older and had more comorbidities than those infected with non-ST81 isolates. There were less serious clinical symptoms in patients infected with ST81 isolates than in the non-ST81 group. ST81 genotype’s lower toxin production would explain the less serious clinical symptoms in ST81 infected patients. We hypothesized that the outbreak of *C. difficile* ST81 infection was due to a combination of the strain’s low virulence and high antibiotic resistance, as well as the immune-compromised status of elderly hosts with comorbidities.

In conclusion, our study presents the first epidemiological evidence in a general teaching hospital in China of an outbreak of epidemic *C. difficile* genotype ST81 by whole genome sequencing in older patients with multiple comorbidities. Continuous surveillance is warranted to reveal the dynamics of ST81 transmission among hospitalized patients. All genotypes, especially hypervirulent strains RT027 should be closely monitored.

## Materials and Methods

### Study design and strains collection

The study was carried out in a 1400-bed general teaching hospital in Shanghai, China between May 2014 and August 2015. All patients diagnosed with suspected CDI were tested for the presence of *C. difficile*. Suspected CDI patients were those with diarrhea (the passage of three or more unformed or loose stools per day for more than three days) without taking laxatives or an enema^25^. Stool samples of the suspected patients were sent to the clinical microbiology laboratory and inoculated onto *C. difficile* selective medium CDIF (BioMerieux, France) in an anaerobic environment at 37 °C for 48 hours. Suspected isolates were identified by API 20 A (BioMerieux, France) and MALDI-TOF MS. Common diarrhea pathogens, such as *Salmonella*, *Shigella, S.aureus*, and *Escherichia coli*, were also detected by culture. Fecal samples were cultured directly on MacConkey agar (Oxoid Ltd, Basingstoke, UK), *Salmonella-Shigella* (SS) agar (for *Salmonella* and *Shigella*) and blood plate (for *S.aureus* and *E.coli*) incubated overnight at 37 °C. Suspected isolates were identified by MALDI-TOF MS. The collection of bacterial isolates from patient specimens and use of the related clinical information of patients were approved by the ethics committee of Renji Hospital, School of Medicine, Shanghai Jiaotong University, Shanghai, China (Reference number: [2016] N003).The requirement for informed consents was exempted as the study was a retrospective chart review. Patient records/information was analyzed anonymously prior to analysis. Duplicate specimens from the same patients were excluded. *C.diffcile* isolates were maintained in MICROBANK (PRO-LAB, Canada) at −80 °C for long term storage.

### Definitions and data collection

A diagnosis of CDI was made according to first verification of toxigenic *C. difficile* isolates in diarrheal stool. All toxin-positive isolates were confirmed by toxin gene detection (as described below). Severe diarrhea was defined as bloody diarrhea and/or diarrhea with hypovolemia or hypoalbuminemia (albumin level < 20 g/L), fever (temperature > 38 °C) and leukocytosis (white blood cell, WBC > 12 × 10^9^ cells/L), and/or pseudomembranous colitis^26^. An outbreak was defined when the number of isolates with the same genotype in a month exceeded double the monthly average^27^. Patient-level information was extracted from patients’ electronic medical records and comprehensively reviewed. This included inpatient demographics, period of hospitalization, comorbidities (using the Charlson index, a well-validated mortality prognosis index)^28^, medication use (including history of antibiotic and glucocorticoid drug use), as well as several laboratory findings, such as conventional stool and occult blood, WBC count, serum albumin, serum creatinine, and serum total bile acid. We examined all antibiotic prescriptions written during hospitalization but excluded exposure to metronidazole and oral vancomycin as these may have been treatments for CDI. According to mechanism and antimicrobial spectrum, antibiotics were categorized into five groups: clindamycin, fluoroquinolones, penicillin derivatives, cephalosporins, and macrolides. Clindamycin and macrolides were infrequently prescribed (2% of all orders). Several laboratory parameters (WBC, conventional stool and occult blood, WBC count, serum albumin, serum creatinine) were extracted from laboratory records as the lab value nearest to and at a maximum of 3 days before the date of the positive stool test result^29^. Serum total bile acid were extracted at a maximum of 10 days before the date of the positive stool test result.

### MLST

Multi-locus sequence typing (MLST) was performed and analyzed according to the method from previous publication^30^. *Adk*, *atpA*, *dxr*, *glyA*, *recA*, *sodA* and *tpi* were amplified and sequenced and results submitted to the MLST database (http://pubmlst.org/ *clostridium difficile*) to obtain the allele profile and sequence type (ST). The eBURST algorithm (http://eburst.mlst.net/) was used to infer the evolutionary relatedness of the STs.

### Detection of *C. difficile* toxins

PCR assays for toxin genes (*tcdB*, *tcdA*, *ctdA* and *ctdB*) were performed using primers according to previous study^20, 31, 32^. The total toxin production was quantitatively evaluated via enzyme immunoassays using the VIDAS *C*. *difficile T*oxin A & Bassay (BioMerieux, France)^11^. Vegetable cells were inoculated in brain heart infusion (BHIS) at 72 hours at 37 °C in an anaerobic environment. The culture samples was adjusted to OD600 = 0.3 and centrifuged. The supernatant (300 μL) was transferred to the sample well according to the manufacturer’s instructions. The concentration of the total A and B toxins was proportioned to the final fluorescence intensity.

### Sporulation capacity determination

The experiment to measure the sporulation ability was conducted as described previously with some modifications^11, 33^. *C. difficile* isolates cultured in BHIS broth were incubated for 48 hours and adjusted to approximately OD600 = 1. One-milliliter samples were removed and heated for 25 minutes at 60 °C to induce the production of spores. Appropriate dilutions (10^−1^, 10^−2^, 10^−3^ and 10^−4^) were plated onto the BHIS plates with 2% agar and 0.1% taurocholate (Sangon Biotech) in order to obtain CFU. The heat-resistant cells were counted after 24 hours of anaerobic incubation; acount between 30 and 300 on each plate was considered valid.

### Antibiotic susceptibility tests

Eight antibiotics, chloramphenicol, ampicillin, tetracycline, vancomycin, metronidazole, moxifloxacin, meropenem, and clindamycin, were used to test the minimum inhibition concentration (MIC) for *C. difficile* by agar dilution method according to Clinical and Laboratory Standards Institute (CLSI) guidelines [M11-A8]. ATCC 70057 was used as control. The breakpoints for each antibiotic were based on CLSI recommendations for anaerobes[M100-S25]. The resistance breakpoints determined by the European Committee on Antimicrobial Susceptibility Testing (EUCAST) for vancomycin (>2 mg/L; clinical breakpoints bacteria v5.0; http://www.eucast.org/clinical_breakpoints/) were used in the absence of a CLSI recommendation for vancomycin.

### Whole-Genome Sequencing and Analysis

Genomic DNA from overnight cultures of *C. difficile* was extracted using the Gentra Purgene Yeast/Bact. Kit (Qiagen, Germany), according to the manufacturer’s instructions. The genome sequence of *C. difficile* was sequenced by Shanghai Majorbio Bio-Pharm Technology Co., Ltd. (Shanghai, China) according to Illumina instructions generating Nextera XT paired-end libraries (2 × 250 bp). The average fragment sizes for the pair-end was 300 bp, respectively. The read length was 101 bp. Low quality sequence data were cut and then the reads were assembled using the SOAPdenove v2.04 program (including GapCloser v1.12) (http://soap.genomics.org.cn/)^34^. The analysis of the ST81 cluster genomes was performed using a cluster-specific genome—that is, one environmental isolate with high quality was de- novo assembled using CLC Genomics Workbench and subsequently used as reference. Sequence data were deposited in the National Center for Biotechnology Information under study accession number: SRP102327.

### Epidemiologic investigation of outbreak of *C. difficile* infection

Patient-level information collected included ward information and movement through the hospital. Based on the hospital ward–based transmission model, networks of patients and potential transmission events were constructed for same ST. Potential epidemiological events were inferred when these pairs shared time on the same ward. Most of the putative transmissions identified occurred shortly (<1 wk) after the onset of symptoms, with few >8 wk. Most incubation periods were <4 wk, with few >12 wk^23^. According their criteria we found there was a potential transmission. Simultaneous, environmental sampling from the emergency observation room was performed in a systematic manner (5 × 20,100 cm^2^ areas) in June 1, 2015 with sterile cotton wool swabs moistened with 0.9% normal saline and then cultured immediately for *C. difficile*. Sites commonly associated with the hands of patients, doctors, nurses, visitors, and health care workers were sampled, including sink faucets, door handles, floors, toilets, commodes, bedrails, bedside tables, telephones, and call buttons. Environment samples were directly inoculated onto *C. difficile* selective medium CDIF (BioMerieux, France) in an anaerobic environment at 37 °C for 48 hours, then suspected strains were identified by API 20 A and MALDI-TOF MS.

### Statistical analysis

Statistical analyses were performed using SPSS Software Version 20.0 (SPSS, USA). Either the two-tailed unpaired Student’s t-test or the Mann–Whitney U-test was used for comparisons of continuous variables with or without a normal distribution. Chi-square and Fisher exact tests were used to compare categorical variables if differences existed among groups.

## Electronic supplementary material

Supplementary Information

Supplementary table S1

Supplementary table S4
