# Supplementary material for: Nosocomial transmission of Clostridium difficile Genotype ST81 in a General Teaching Hospital in China traced by whole genome sequencing
Source: Sci Rep. 2017 Aug 29;7:9627. doi: 10.1038/s41598-017-09878-8 (PMC5575120; doi:10.1038/s41598-017-09878-8)
Supplement: Supplementary file 1 — Supplementary Information [file 41598_2017_9878_MOESM1_ESM.doc]

**Nosocomial transmission of *Clostridium difficile* Genotype ST81 in a General Teaching Hospital** **in China traced by whole genome sequencing**

Juanxiu Qin ^1*^, Yingxin Dai ^1*^，Xiaowei Ma^1^, Yanan Wang ^1^, Qianqian Gao^1^, Huiying Lu^1^, Tianming Li^1^, Hongwei Meng^1^, Qian Liu^1^, Min Li ^1^

1: Department of Laboratory Medicine, Renji Hospital, School of Medicine, Shanghai Jiaotong University, Shanghai, China

^*^These authors contributed equally to this work.
Corresponding author: Min Li

E-mail address: [ruth_limin@126.com](mailto:ruth_limin@126.com)

Postal address: Min Li, Department of Laboratory Medicine, Renji Hospital, School of Medicine, Shanghai Jiaotong University, Shanghai, China

**Supplementary table S1. Demographic data, sequence types (STs), toxin genotypes in the 91clinical isolates.**

**Supplementary table S2. STs and Antibiotic Resistance Patterns in 80 Toxigenic Isolates**

| **Genotypes(No.)** | **Resistant patterns (% Resistance)** | | | | | | | |
| --- | --- | --- | --- | --- | --- | --- | --- | --- |
|  | **CLI** | **MOX** | **TET** | **MET** | **VAN** | **CHL** | **MER** | **AMP** |
| **ST81(28)** | **71.4** | **67.9** | **17.9** | **0** | **0** | **0** | **0** | **0** |
| **ST2(9)** | **2^a^** | **1** | **0** | **0** | **0** | **0** | **0** | **0** |
| **ST54(8)** | **100** | **0** | **0** | **0** | **0** | **0** | **0** | **0** |
| **ST129(8)** | **100** | **3** | **0** | **0** | **0** | **0** | **0** | **0** |
| **ST3(6)** | **2** | **1** | **0** | **0** | **0** | **0** | **0** | **0** |
| **ST35(3)** | **2** | **0** | **0** | **0** | **0** | **0** | **0** | **0** |
| **ST98(3)** | **0** | **0** | **0** | **1** | **0** | **0** | **0** | **0** |
| **ST319** | **0** | **0** | **0** | **0** | **0** | **0** | **0** | **0** |
| **Others(14)** | **3** | **2** | **0** | **0** | **0** | **0** | **0** | **0** |
| **Total (80)** | **56.3** | **32.5** | **6.3** | **1** | **0** | **0** | **0** | **0** |

CLI: clindamycin; MOX: moxifloxacin; TET: tetracycline; MET: metronidazole; VAN: vancomycin; CHL: chloramphenicol; AMP: ampicillin; MER: meropenem.

**^a^：**STs with less than 5 drug-resistant isolates for one kind of drug were not calculated in the percentage of antibiotic resistance.

**Supplementary Table S3. Demographic characteristics of excluded and included patients**

| **Variable** | **Excluded patients**  **(n=21)^a^** | **Included patients**  (n=59) | p value |
| --- | --- | --- | --- |
| Age(years;mean±standard deviation[SD]) | 60.29±10.7 | 56.08±21.7 | 0.40^b^ |
| Gender(n[%]) |  |  | 0.07^c^ |
| Male | 15(75.0) | 30(50.8) | - |
| Females | 5(25) | 29(49.2) | - |
| ST(ST[n]) | ST129(7),ST2(5),  ST81(3), others(6) | ST81(25),ST54(7),  ST3(5),ST2(4),others(18) | - |

^a^ :One patient's clinical information is missing；^b^: the two-tailed unpaired Student’s t-test; c: Chi-square.

**Supplementary table S4. Clinical information of hospital patients infected with ST81 and non-ST81 *C. difficile.***

**Supplementary table S5. Primers used in this study.**

| **Primer** | **Gene** | **Sequence（5^，^-3^，^）** | **Fragment size(bp)** |
| --- | --- | --- | --- |
| adk-F | *adk* | TTACTTGGACCTCCAGGTGC | 635 |
| adk-R |  | TTTCCACTTCCTAAGGCTGC |  |
| atpA-F | *atpA* | TGATGATTTAAGTAAACAAGCTG | 674 |
| atpA-R |  | AATCATGAGTGAAGTCTTCTCC |  |
| dxr-F | *dxr* | GCTACTTTCCATTCTATCTG | 525 |
| dxr-R |  | CCAACTCTTTGTGCTATAAA |  |
| glyA-F | *glyA* | ATAGCTGATGAGGTTGGAGC | 625 |
| glyA-R |  | TTCTAGCCTTAGATTCTTCATC |  |
| recA-F | *recA* | CAGTAATGAAATTGGGAGAAGC | 705 |
| recA-R |  | ATTCAGCTTGCTTAAATGGTG |  |
| sodA-F | *sodA* | CCAGTTGTCAATGTATTCATTTC | 585 |
| sodA-R |  | ATAACTTCATTTGCTTTTACACC |  |
| tpiA-F | *tpiA* | ATGAGAAAACCTATAATTGCAG | 640 |
| tpiA-R |  | TTGAAGGTTTAACTTCCACC |  |
| tcdA-F | *TcdA* | AGATTCCTATATTTACATGACAATAT | Positive：369 |
| tcdA-R |  | GTATCAGGCATAAAGTAATATACTTT | Negative：110 |
| NK104(tcdB-F) | *TcdB* | GTGTAGCAATGAAAGTCCAAGTTTACGC | 204 |
| NK105(tcdB-R) |  | CACTTAGCTCTTTGATTGCTGCACCT |  |
| cdtB-F | *cdtB* | CTTAATGCAAGTAAATACTGAG | 510 |
| cdtB-R |  | AACGGATCTCTTGCTTCAGTC |  |
| cdtA-F | *cdtA* | TGAACCTGGAAAAGGTGATG | 375 |
| cdtA-R |  | AGGATTATTTACTGGACCATTTG |  |
| 16S | *16-23srDNA* | GTGCGGCTGGATCACCTCCT |  |
| 23S |  | CCCTGCACCCTTAATAACTTGACC |  |

**Supplementary Figure S1****.** **Monthly distribution of Antibiotics Use Density (AUD) for quinolones in the emergency department** (AUD= DDD/100 bed-days; DDD: Defined Daily Dose)

**
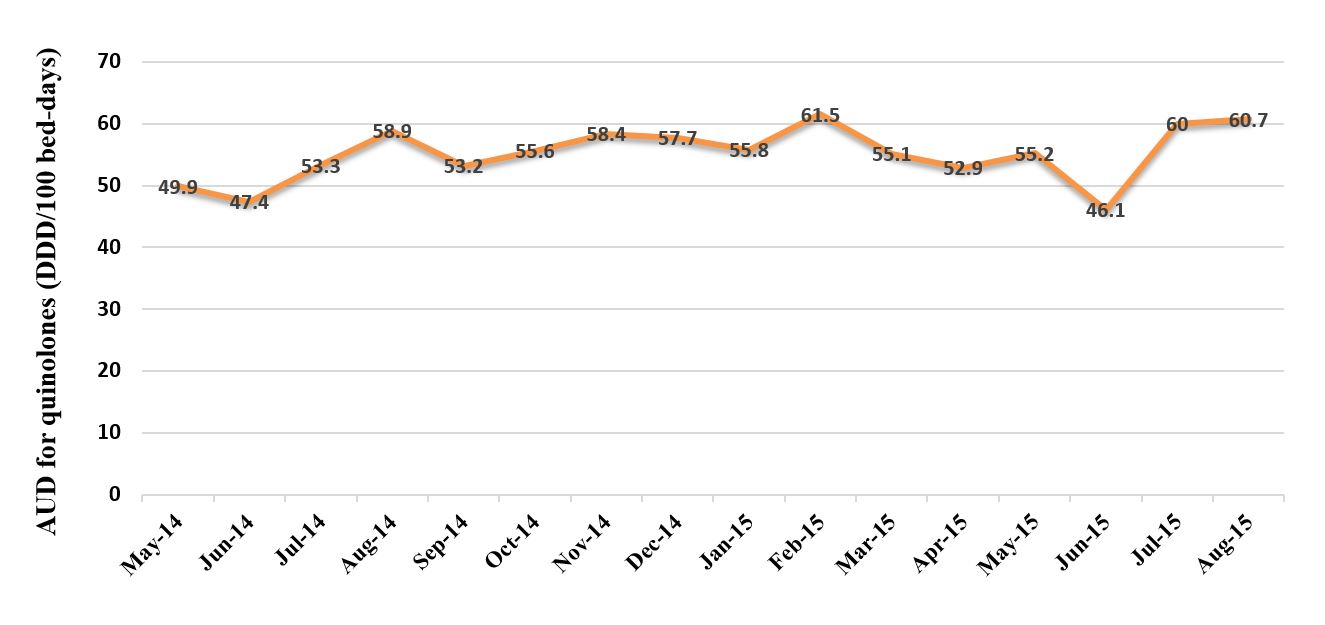
**
